# Validation of a novel simulated tendon model for core suture tendon repair

**DOI:** 10.1308/rcsann.2024.0064

**Published:** 2024-09-24

**Authors:** L Western, PG Roberts, J Rees, D Howgate

**Affiliations:** University of Oxford, UK

**Keywords:** Tendon D013710, Simulation Training D000066908, Silicones D012828, Education Q000193

## Abstract

**Introduction:**

Simulation training can develop surgical procedural skills in a safe environment. Able to offer high-intensity exposure, simulation is increasingly important as working time for surgeons becomes more protected. Materials used in simulated tendon repair play a critical role in the fidelity and face validity of the model. Although organic materials like porcine tendon are commonly used, non-organic materials offer advantages such as accessibility, reproducibility, cost-effectiveness and ease of use without the need for special licences or facilities. This study aims to establish the face, content and concurrent validity of using a novel silicone material in a simulated tendon repair model.

**Methods:**

Three tendon models, bathroom silicone sealant, DragonSkin^®^ silicone and organic porcine tendons, were evaluated for concurrent validity through mechanical load to failure testing. Face and content validity were assessed, following participant repair of a DragonSkin^®^ tendon, using a 5-point Likert scale for five clinically relevant parameters.

**Results:**

Significant differences in load to failure were observed among bathroom sealant, DragonSkin^®^ and porcine tendon (11.1N, 31.7N and 56.2N; *p *< 0.001). Participant feedback on the DragonSkin^®^ tendon indicated that it was suitably representative, easy to use and useful for training (agreement rates 58%, 75% and 83%, respectively). However, participants noted that the model did not handle or glide like human tendon (both 8% agreement).

**Conclusion:**

DragonSkin^®^ silicone is an adaptable and valid material for simulated tendon repair models. It is low cost, widely available and shows promise as a training tool. Future research will focus on exploring its effectiveness in training settings.

## Introduction

Tendon repair is an index procedure in both orthopaedic and plastic surgical specialty training curricula.^1,2^ Focused and task-specific simulation training provides the opportunity for trainees to learn, practise and develop defined psychomotor surgical skills in a controlled environment outside the confines, time constraints, pressure and risk of a clinical setting. Several studies have attempted to develop simulated tendon repair models, but no consensus has been reached on the optimal material to use in this scenario.^2–5^ Considerable variations exist in the cost, fidelity, feasibility and level of evidence supporting the use of specific materials in surgical simulators. The concept of fidelity in relation to simulation refers to how realistic the simulation model appears to the learner (physical fidelity) and whether the task replicates what is expected in real-world performance (functional fidelity). In general, the fidelity of a simulator should match the complexity of the procedure or task to be replicated.^4,5^

The materials used in simulated tendon repair models may be broadly classified as organic or non-organic. Organic materials such as animal or human cadaveric tissue possess the highest levels of physical and functional fidelity, and for this reason have been considered the gold standard for use in surgical simulation training.^2,6^ However, organic tissue is expensive, carries a risk of transmissible diseases, and within the United Kingdom (UK), a special licence from the Home Office is required for using human tissue.^2,6–8^ The use of human cadaveric specimens in surgical simulation training also raises ethical considerations and is limited to well-funded and licensed institutions with appropriate access to specialised facilities. The condition of cadaveric specimens may also vary dependent upon the preparation method used (fresh frozen or embalmed), and the quality of tissues will degrade with repeated use. Furthermore, the consistency in anatomy may vary between specimens, and specific pathological conditions of interest may not be present. These factors may reduce the reproducibility in user experience in both cadaveric and animal tissue simulation models.^6^

A range of non-organic materials have been used in simulated tendon repair, including rope, liquorice, catheters and bathroom sealant.^2,3,7,9,10^ These materials provide logistical and ethical advantages but are generally considered to possess lower physical and functional fidelity than organic materials. The benefits of using non-organic materials are that they are relatively easily accessible, reproducible, cost-effective and do not require any special licences or facilities to use.

Materials used in simulation training should possess face and content validity. Face validity describes how realistic the material or simulation model is. Content validity is the extent to which the simulation model is able to accurately assess or represent the skills and knowledge that it purports to measure (technical surgical skills).^11^ Concurrent validity describes how well the material or simulation model performs in comparison with other materials or models. One study reported that silicone bathroom sealant holds similar face and content validity as porcine tendon in a simulated human tendon repair model and that it exceeds that of basic non-organic materials such as liquorice.^2^ However, the authors of this study experienced potential mechanical drawbacks with the use of sealant, including cutting through of suture material when tying the knots. Another potential challenge with using silicone sealant is the need to expose this material to air during the curing process. This makes injection moulding and the creation of consistent custom shapes challenging. DragonSkin^®^ (Smooth-On, Macungie, PA, USA) is a premium precipitant silicone that has been used for knee arthroscopy simulation models.^12^ The product is similar in price to silicone sealant, but has improved physical properties and may be a more robust material for tendon repair models.^13^

The aim of this study is to establish the face, content and concurrent validity of using this DragonSkin^®^ material in a simulated tendon repair model.

## Methods

### Material preparation

**Figure 1:**
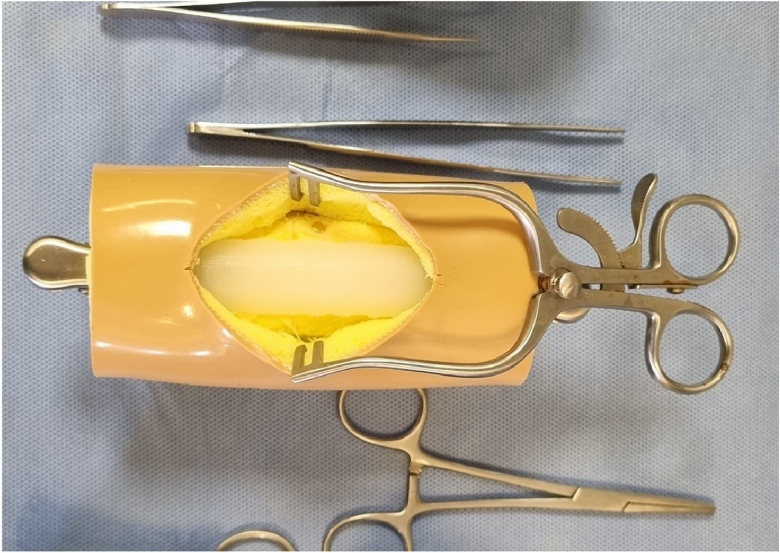
A model Achilles tendon was cast in a custom mould. The simulation rig was created with wood, foam and plastic. Clips mounted to the rig hold the tendon in place during the simulation exercise.

DragonSkin^®^ was prepared by mixing the two precipitant liquids (platinum cure silicone rubber and silicone rubber compound) under vacuum and casting into an 8mm tube.^13^ Silicone sealant (No-Nonsense silicone white sealant, Kingfisher International Products Ltd, London, UK) was piped freely from an 8mm nozzle to form cylindrical simulated tendon. Porcine tendons were harvested from pig trotters utilising a previously described technique.^8^ Each tendon material was cut to 9cm in length, then sharply divided mid-substance into two equal 4.5cm segments. These segments were then repaired utilising a 3:0 Ethibond suture material with a two-strand modified Kessler technique by a single surgeon experienced with this technique (Figure 1). Tendons undergoing tensile loading had their diameters measured and confirmed using a digital calliper.

### Mechanical load to failure

**Figure 2:**
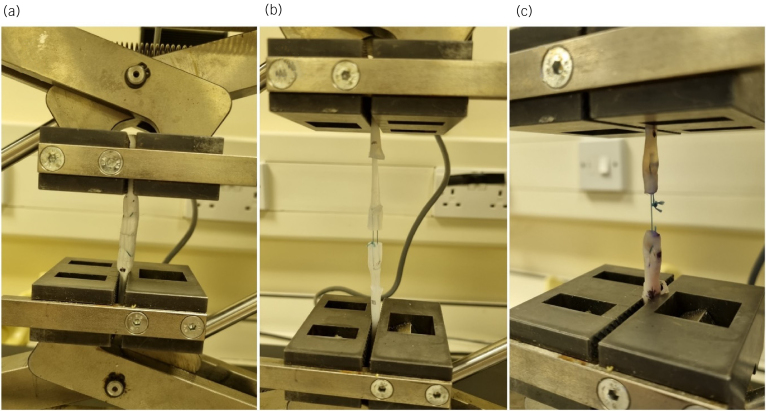
Load testing of the organic and non-organic materials: (a) demonstrates sealant silicone at preload 0.5N; (b) demonstrates DragonSkin^®^ silicone at failure via tendon rupture; and (c) demonstrates porcine tendon at failure via suture rupture.

To determine concurrent validity of DragonSkin^®^, its mechanical properties were compared with silicone sealant and porcine tendon models by mechanical load to failure testing. Tensile loading was performed using a universal testing device (Z005 Zwick/Roell GmbH, Ulm, Germany). Tendons were mounted at each end by metal gripping clamps 2cm from the midline repair. Tendons were preloaded to a force of 0.5 Newtons (N) then loaded continuously at a rate of 20mm/min until failure.^14,15^ The load to failure and the mechanism of failure was recorded. Any slippage of tendon material would invalidate a result (Figure 2).

**Figure 3:**
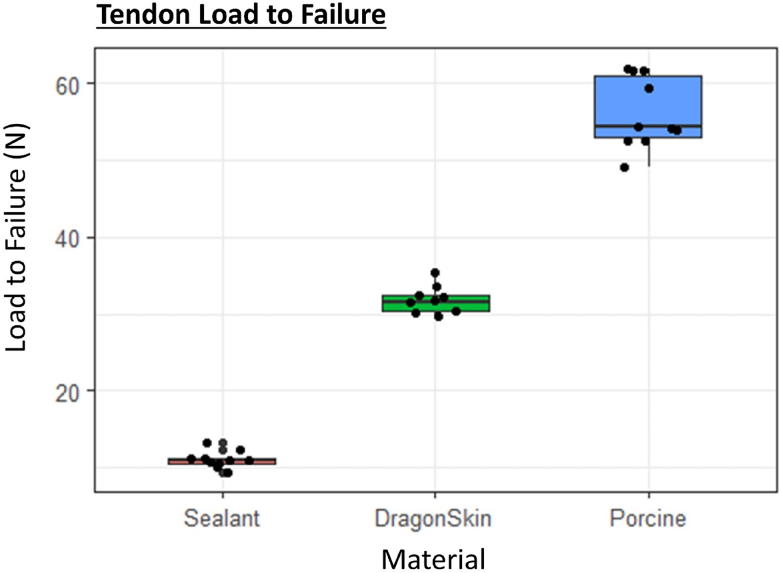
A box and whisker plot with interquartile range illustrating the load to failure in newtons (N) for each material. Black dots represent individual data points.

### Expert participant feedback

Participants in recognised higher surgical training programmes or consultants with operative experience in tendon repair were invited to participate in this study. Feedback was collated via Google Forms (Google Forms; Google LLC, Mountain View, CA, USA). Five parameters were assessed using a 5-point Likert scale.^2^ These parameters were: grading the tendon appearance, handling, gliding, ease of use and value for training. Free-text space was provided for participants to comment on the perceived advantages and disadvantages of this material. Qualitative themes of comments were established utilising an emergent thematic approach. Likert scale results and qualitative findings are compared to achieve conclusions.

### Statistical analysis

All participants involved in the study provided written consent for their anonymous data to be analysed for the purposes of this study. Statistical analysis and graphical visualisation were performed using RStudio (PBC, Boston, MA, USA). Normality of data distribution was not assumed, and non-parametric statistical tests were used. Load to failure was measured in newtons and the Kruskal–Wallis test was used to determine any statistically significant groupwise differences. The interrater reliability of the ordinal Likert scale was calculated utilising a two-way random intraclass corelation coefficient analysis (ICC). The significance threshold was set at 0.05 for all statistical analysis.

### Ethical approval

Ethical approval was obtained through the Oxford University Ethics Committee (MSD-IDREC R57808/RE001).

## Results

### Load to failure

Ten samples of each material were mechanically loaded to failure. There were no slippages or failed tests. Porcine tendon was the most resistant to load, and sealant the least. There were differences in the mechanism of failure between materials (Table 1). Significant differences in load to failure were observed among bathroom sealant, DragonSkin^®^ and porcine tendon (Mean value of 11.1N, 31.7N and 56.2N respectively; *p* < 0.001) (Figure 3). Post-hoc Dunn’s test with Benjamini–Hochberg method correction demonstrated highly significant differences between each group (*p *< 0.02 for all).

**Table 1:** Results of the load to failure testing

Material	Diameter (mm) [sd]	Load to failure (N) [sd]	Cut-through (%)	Tendon rupture (%)	Suture rupture (%)
Sealant	8.5 [0.7]	11.1 [1.8]	60	40	0
DragonSkin^®^	8 [0]	31.7 [1.1]	0	100	0
Porcine	7.6 [0.5]	56.2 [4.6]	0	0	100

Mean and standard deviation [sd] of diameter of materials at the cut end and the load to failure in newtons (N) are indicated. The mechanism of failure is recorded as a percentage of cut-though, tendon rupture or suture rupture.

### Participant feedback

Twelve participants specialising in plastic or orthopaedic surgery completed the tendon repair task, including specialty registrars (UK equivalent to resident in the USA, *n *= 7), an orthopaedic fellow (*n *= 1) and consultants (*n *= 4). The ICC of 0.91 (95% CI 0.77–0.98) indicates excellent reliability of the participant feedback responses.

Most participants agreed that the tendon model looked representative, was easy to use and could be a useful tool for training. There was general disagreement that the tendon handled or glided well in comparison with human tendon (Table 2).

**Table 2:** Response to each question indicated as a cumulative total of participants rating at each Likert scale

Likert response	Strongly disagree	Disagree	Neutral	Agree	Strongly agree	Overallagreement (%)
Looks representative of tendon repairprocedure	0	2	3	5	2	58
Handles like human tendon	1	6	4	0	1	8
Glides like human tendon	2	7	2	0	1	8
Simulation is easy to use	0	0	3	4	5	75
Simulation is useful for training	0	1	1	6	4	83

Overall agreement is the percentage of participants who rated the question with ‘agree’ or ‘strongly agree’

A similar consensus can be drawn from the qualitative data. Positive themes included representativeness, good tool for practising surgical technique and that it is useful for learning relevant instrument handing:

• “Anatomically representative”.

• “This is all about practising the suture pattern and getting it practised. I think it’s well worth training doctors with this”.

• “Good for going over basic instrument handling in relation to tendon repair”.

Negative themes included issues around the suture gliding through the material, sensation of the material being too stiff and some surgeons altering their surgical technique:

• “Need to pull the distal loops through sequentially in order to pull the gap together”.

• “Does not have the consistency of human tissue”.

• “Too stiff”.

• “Was impossible to pick up the edge of the tendon with forceps … you have to hold the whole tendon to do the exercise”.

## Discussion

The results of this study support the face, content and concurrent validity of DragonSkin^®^ silicone as a simulated tendon repair model. The load to failure of DragonSkin^®^ silicone is more representative of animal tendon than regular silicone sealant, which has been recommended in previous research.^2^ Furthermore, the mechanism of failure for DragonSkin^®^ is primarily tendon rupture, rather than suture cut-through. This has advantages because participants can tighten surgical knots without concern of the suture cutting out of the material as easily as with bathroom sealant. Adequate tensioning of the suture in real tendon repair reduces the risk of tendon ‘gapping’ once repaired. Minimal gapping is important for tendon repair outcomes, and thus it is important that techniques that can reduce gapping can be effectively practised in simulation models.^15,16^

DragonSkin^®^ appears to have mechanical and logistical advantages over other materials reportedly used in simulated tendon repair models.^2,3,7,9,10^ One major advantage of DragonSkin^®^ in comparison with standard silicone sealant is that it can be injection moulded into any shape and cured without exposure to air. This will allow a wide variety of moulds to be produced with consistency and uniformity in the simulation models. Moulding may also allow users to create custom anatomical tendon simulation models that are relevant to their subspecialty interest. This would be more difficult with other non-organic material suggestions, such as catheters, or piping sealant, which generally restrict users to cylindrical simulated tendon models.

The DragonSkin^®^ silicone model provides face validity, with a majority in agreement that it appeared anatomically representative (Table 2). Participant feedback demonstrated strong agreement that the simulation was useful for practising tendon repair and instrument technique, which supports the content validity of this model. Excellent inter-rater reliability for the feedback questionnaire responses adds further strength to this finding. However, participants reported that the DragonSkin^®^ tendon model did not handle the sutures, or glide, like human tendon. This may have implications in the acquisition of fine motor skills of passing suture. A previous study found participant feedback from seven professionals was generally positive of bathroom sealant for gliding and handling (100% and 71% in agreement respectively).^2^ It may be that the denser DragonSkin^®^ silicone provides more friction to the suture material than silicone sealant, thus impairing the gliding and handling sensation. It is possible that utilising monofilament sutures, wetting sutures or alternative DragonSkin^®^ preparations, which are less dense, may improve gliding sensation if a user feels these issues detract from the simulation quality, but this has not been evaluated in this study.^13^ However, the mechanical benefits of DragonSkin^®^ silicone allow the application of more force, which will enable participants to practise knot-tying skills effectively, without danger of cutting through the tendon material (Table 1). Despite these model limitations content validity was maintained, with participants finding the DragonSkin^®^ silicone easy to use and a useful model for training tendon repair (Table 2).

### Study limitations

A limitation of this study is that the DragonSkin^®^ silicone model was reviewed in isolation by the participating surgeons. Although the biomechanical properties of simulated tendon models using DragonSkin^®^ were assessed in comparison with silicone and porcine animal tendons, the user experience was not. Future research may explore this in further detail in comparison with other commonly used materials in simulated tendon repair models.^2,3^

## Conclusions

A precipitant silicone, DragonSkin^®^, is a low-cost and valid material for use in simulated tendon repair models. It handles sutures more robustly than bathroom silicone sealant and provides additional benefits in moulding and curing bespoke shapes used in simulated tendon models. Non-organic materials provide practical solutions to the drawbacks experienced with animal tissue for integration into simulation training.^2,3^ Future research should investigate the construct validity of this simulation model to establish its role as an effective educational platform for tendon repair.
